# SMA-Driven Assistive Hand for Rehabilitation Therapy

**DOI:** 10.3390/s25216782

**Published:** 2025-11-05

**Authors:** Grace Mayhead, Megan Rook, Rosario Turner, Owen Walker, Nabila Naz, Soumya K. Manna

**Affiliations:** School of Sciences, Psychology, Arts and Humanities, Computing, Engineering & Sports, Canterbury Christ Church University, Canterbury CT1 1QU, UK; g.mayhead507@canterbury.ac.uk (G.M.); m.rook742@canterbury.ac.uk (M.R.); r.turner455@canterbury.ac.uk (R.T.); o.walker83@canterbury.ac.uk (O.W.); nabila.naz@canterbury.ac.uk (N.N.)

**Keywords:** assistive device, shape memory alloy, thermal management, rehabilitation therapy, mirror training

## Abstract

Home-based rehabilitation supports neuromuscular patients while minimising the need for extensive clinical supervision. Due to a growing number of stroke survivors, this approach appears to be more practical for patients across diverse demographics. Although existing hardware-based assistive devices are pretty common, they have limitations in terms of usability, wearability, and safety, as well as other technical constraints such as bulkiness and torque-to-weight ratios. To overcome these issues, soft actuator–based assistance prioritises user safety and ergonomics, as it is more wearable and lightweight, offering overall support while reducing the social stigma associated with disability. Among the existing soft actuation techniques and related materials, shape memory alloys (SMA) present a feasible option, offering current-controlled actuation and compatibility with integration into flexible textiles, thereby enhancing the wearability of the device. This paper presents a compact, SMA-driven assistive device designed to support natural motion, reduce muscle fatigue, and enable daily therapy. Embedded in a glove, the device allows mirrored control, where one hand’s movement assists the other, using flex sensors for feedback. The functionality of the electromyography (EMG) sensor is also used to evaluate the activation of the SMA wire; however, it is not employed for detecting individual finger motions in the assistive hand. Polyurethane foam insulation minimises thermal effects while maintaining lightweight wearability. Experimental results demonstrate a reduction in actuation time at higher voltages and the effective lifting of light to moderate weights. The device shows strong potential for affordable, home-based rehabilitation and everyday assistance.

## 1. Introduction

In the UK, around 100,000 people suffer from stroke annually, while 1.3 million survivors are currently living with it [1]. Stroke-related hand impairment often affects motor function. A large number of patients deal with arm weakness and lack of strength in hand movement and gripping after a stroke [2]. It is crucial to undergo effective rehabilitation following a stroke to enhance recovery of joint movement and muscle strength. Targeted and repetitive biomechanical exercises, supported by technology, will contribute to stroke-related recovery [3]. Compared to standard rehabilitation practices, where physiotherapists assist patients physically through passive joint movement to achieve the anatomical range of motion in a specific direction, technology-assisted methods, such as assistive devices, guide patients with more accurate and error-free joint movements, eliminating fatigue. These exercises are designed to improve neuromotor functions, which indirectly affect muscle spasticity, strength and joint movement [4]. These assistive devices not only manage to provide consistent, controlled, and systematic supportive movements but also can monitor the performance of users and recovery progress throughout the process [5]. To stimulate neuroplasticity and enable the brain to regain neural pathways, repetitive motion is essential. Patients can recover their physical abilities which was lost to stroke-related tissue damage through rigorous and repetitive training [6].

While there are limiting factors of hardware-based motorised devices, such as bulky design, mechanical appearance, weight, poor ergonomics, safety issues, and the social stigma associated with visibly assistive equipment [7], soft actuator-based devices are gaining popularity as they provide compliant and safe joint movements, improved wearability, and can be integrated into garments. There are various types of materials used to develop soft robotic hands, including elastomers, silicone, and flexible plastics, which are fabricated through moulding [8] and other additive manufacturing techniques, such as 3D printing [9]. Among existing soft actuators, shape memory alloys (SMA) [10], electroactive polymers (EAP) [11], and pneumatic muscles [12] appear widely in research studies. Pneumatic muscles are based on pneumatic actuation, which usually depends on a tethered air compressor; their use in portable systems remains challenging. Pneumatic actuators also exhibit nonlinear behaviour, while EAPs are highly energy-consuming.

Shape memory alloys (SMAs) is increasingly recognised as promising actuators for prosthetic and rehabilitative applications due to their lightweight nature, biocompatibility, silent operation, and ability to mimic natural muscle contraction. Their flexibility makes them particularly well-suited for rehabilitation devices, offering comfort and adaptability in medical applications. The biomimetic nature of SMAs allows them to function similarly to human muscles, contracting in response to external stimuli such as heat. Among SMA types, nickel–titanium (NiTi or Nitinol) wires and coiled SMA actuators are the most prevalent in recent designs. SMA have two phases: austenite, the stiff high-temperature phase, and martensite, the flexible low-temperature phase. When heated, martensite transforms back into austenite, regaining its original shape, ideal for controlled movement in assistive hand devices [13]. The structural formation of SMA typically undergoes a two-phase transformation: austenite and martensite, depending on the temperature provided or the controlling current. When heated, martensite reverts to austenite, restoring its original shape and making it suitable for controlled movement in assistive hand devices [13]. The austenite phase for NiTi-based SMAs starts at 8.5 °C and ends at 33.9 °C, whereas the martensite phase begins at −41.18 °C and concludes at −64.13 °C [14]. These transition temperatures allow for precise thermal activation and control of movement. Research efforts focus on enhancing actuation performance, specifically by optimising force generation, cycle speed, and control precision. At the same time, commercial rehabilitation devices prioritise affordability, ease of use, and therapeutic outcomes over advanced actuation technologies. A comprehensive survey is explored, including both research-driven SMA devices and commercially available assistive systems, highlighting their strengths and limitations.

### 1.1. Actuator Types and Characteristics

I. Wire-based SMA actuators are made from NiTi alloys and are widely used in prosthetic and robotic hands. These lightweight actuators can generate muscle-like motion by contracting in response to heat or an electric current. Wire-based systems are simple and can adapt to different finger and joint configurations, making them a logical option for anthropomorphic prosthetic designs [15].
II. Coiled SMA actuators are shaped into spring-like forms to improve displacement and force output. This configuration allows for larger strokes and greater actuation efficiency, which are advantageous for soft robotic hands made using additive processes like thermoplastic polyurethane (TPU) 3D printing [16].
III. Linear SMA actuator based on Nitinol. Researchers have shown that grip force and actuation speed can be significantly enhanced compared to traditional servo-driven prosthetic hands by incorporating two-link couplings [17].
IV. In composite SMA actuators, SMA elements are combined with other materials to develop a smart composite that improves cooling rates, structural stiffness, and overall performance [18] compared to their original properties.

### 1.2. Prosthetic and Robotic Hands

Thorough market research is conducted to explore existing devices that utilise SMA for assistance. The initial advancements in SMA-driven prosthetics focused on tendon-driven mechanism. For example, a novel anthropomorphic hand controlled by antagonistic pairs of SMA wires used a tendon-driven mechanism to provide three active degrees of freedom (DOF), demonstrating the feasibility of SMA-driven tendon systems for prosthetics [19]. The device has been effective in prosthetic and rehabilitative applications. Tendon-driven SMA could mimic natural finger movements while being worn by users. Another underactuated tendon-driven prosthetic hand, controlled by SMA wires, has integrated tactile sensors into its fingertips [20]. The hand’s small size, lightweight structure, and quiet operation made it especially promising for daily use by individuals with upper limb amputations. Another example is a low-cost prosthetic hand that replaced conventional SG90 servomotors with Nitinol-based linear SMA actuators [17].

Additive manufacturing is utilized in combination with SMA coiling for producing customisable and affordable prosthetic devices. A fully 3D-printed hand is crafted from thermoplastic polyurethane (TPU) and powered by custom-coiled SMA springs [16]. A soft, five-fingered hand is also developed using 100 μm SMA wires. To generate higher output forces, multiple SMA wires were bundled together, producing a silent, flexible, and customisable robotic hand [21]. Their study evaluated the displacement of individual phalanges and examined the hand’s dynamic responsiveness to various power inputs, highlighting its suitability for grasping a wide range of objects. Researchers have investigated glove-like exoskeletons using SMA actuators, which encompass a lightweight system that assists wrist movement [22] and soft exoskeletons [23]. A diverse range of sensors has been embedded into these assistive devices to act as a feedback channel and for controlling movements. Tendon-based assistive systems driven by SMA have shown effective motion in response to Electromyography (EMG) based control when combined with actuation via pulse-width modulation (PWM) [19]. While resistance-based feedback has facilitated precise position control in tendon-driven designs [20], nonlinear control strategies are still necessary to address the complexities associated with SMA actuation [23]. The performance of these systems is determined by their load capacity and response time, which vary depending on the design. As previously discussed, a 3D-printed hand driven by SMA can lift loads of up to 133 g, although its operating frequency was relatively low (0.125 Hz) [16]. In contrast, linear Nitinol-based actuators generated grip forces that achieved a power grip of 16.6 N, a tripod grip of 7.3 N, and a pinch grip of 4.4 N [17].

There are wearable wrist-assist devices (weighing 152 g) that utilise SMA contractions for joint movement and can produce forces of up to 10 N, contracting up to 40% of their original length, thereby providing effective support for wrist flexion/extension and radial/ulnar deviation [22]. The device weighed only 151 g and produced torques of up to 1.32 Nm, making it comfortable for daily rehabilitation. The heating times for linear Nitinol actuators are minimised, with limits of only 0.06 s and 0.11 s, respectively, and positional accuracy within 1°. This work demonstrated the viability of Nitinol actuators for affordable prostheses in low-resource settings [17]. With their improved grip strength and quick actuation, linear SMA prostheses can serve as effective low-cost options. By bundling SMA wires in a device, it is possible to grasp objects of various shapes and sizes [21], which is supported by resistance-based control. At the same time, tactile sensing is integrated to enhance its precision and usability [20]. Hence, the SMA actuator-based wearable assistive device could be a potential solution for rehabilitation-related movements while enhancing daily life activities [22]. In addition, these devices could enhance user comfort and flexibility compared to rigid motorised systems [23]. A list of SMA-driven assistive hand devices is shown in Table 1.

### 1.3. Commercial Rehabilitation Devices

In contrast to SMA devices, which focus on research, commercial assistive devices prioritise portability, user-friendliness, and therapeutic efficacy over innovations in new types of actuation techniques (Table 2). There are several commercial devices which can deliver rehabilitation as well as assistance for hand movement, for example, the XFT Hand Rehab System, which can provide both functions required for neuromotor recovery, including muscle stimulation integrated with mobility training [25]; TENS Pain Relief + iGlove supports pain alleviation in users utilising preset TENS modes attached to conductive fabric [26]; the SaeboGlide Plus is a guided exercise device used for stroke rehabilitation [27]. Further devices comprise the Radial Nerve Palsy Brace Splint to keep fingers extended and provide wrist assistance during movement [28], the EMFOCU Robotic Rehab Gloves featuring sequential training options through mirror therapy [29], and ixorelia’s Advanced Rehabilitation Robot Glove, which is a smart glove used for mirror treatment [30]. Gear Tech’s Rehabilitation Robot Glove is also a popular device for various therapy modes, controlled with a range of speeds up to nine levels [31]. In contrast, the advantages of the XTENSOR hand exerciser focus on enhancing users’ grip strength and finger movement [32]. Most commercially available assistive and rehabilitative devices are actuated either pneumatically or through passive mechanisms such as springs or elastic bands to avoid the complexities associated with advanced actuation systems. These commercial devices are priced between GBP 38 and GBP 899, indicating accessibility to different consumer segments. Currently, there are no commercially available devices that utilise SMA actuation, indicating that SMA-based solutions remain largely in the research and prototyping stage, rather than in widespread clinical or market use.

Due to the flexibility and durability of conductive textiles, it is suitable for incorporating sensors into wearable devices that conduct electricity, thereby supporting the actuation of SMA wires [33]. These textiles are usually made with conductive fibres, metal coatings, or woven conductive threads. Polyester and Lycra are popular materials used to offer effective support when combined with Nitinol wire, due to their resilience and compatibility [34]. By using Nitinol wire, cotton and wool can be combined to produce a structure with greater elasticity. The design of the wearable glove device [35] incorporates this feature.

### 1.4. Limitations of Existing SMA-Based Actuators

Even with advancements, SMA-based devices still encounter various structural and operational constraints. The efficiency and responsiveness of SMA actuation are limited by nonlinear heating and cooling dynamics, hysteresis, and slow cycle rates [23]. A significant challenge is the limited load-bearing capacity of SMA actuators, which often cannot produce enough force without mechanical amplification and require a relatively high energy input for effective actuation [36]. As the cooling process is slow, this further limits the actuation bandwidth. Hence, such devices are not well-suited for fast and repeated motion. Additionally, the nonlinear and hysteretic nature of SMA behaviour complicates the control algorithm used for joint movement.

While thin SMA wires offer silent and compliant actuation, they lack sufficient strength to generate a significant force for joint assistance without being bundled together, which again increases the weight and complexity of the system [21]. Finally, the lack of comprehensive long-term clinical assessments, especially trials involving amputees or stroke patients, constitutes a major obstacle that hinders the transition of SMA-based devices from experimental prototypes to clinically validated technologies [17,20].

This project aims to develop an SMA-based assistive hand-supporting device to assist individuals with limited motor skills in their left hand, primarily targeting stroke victims but also benefiting those with conditions such as trigger finger, carpal tunnel syndrome, arthritis, and Dupuytren’s disease. These conditions impair hand movement through symptoms such as tendon thickening, numbness, or joint pain [37]

After reviewing the existing limitations of SMA actuation techniques, the proposed device incorporates several design features to enhance support for human joint movement during rehabilitation.

- Assistance: The device is designed to assist finger flexion and extension, ensuring finger motion and load capacity. It has been evaluated through a comparative analysis of the range of motions for each finger joint and hand trajectory which could support mirror therapy in future. Also, a basic load capacity test has been carried out to identify the maximum load under a stable electrical input, and to understand the relationship between the applied load and actuation performance.
- User comfort: SMA actuators are integrated into a glove through a structured framework that maintains wearability and comfort while not interfering with finger movement. It has been evaluated through structural design.
- Thermal management: Polyurethane foam insulation is applied around the SMA surface to minimise heat transfer, thereby improving safety and user comfort, evaluated through a surface temperature study between insulated and non-insulated models.
- Actuation time: Experimental evaluations are conducted across a range of input voltages to optimise and reduce actuation time at operating voltage and evaluated through a comparative activation test at 3.3 V and 5 V (at 5 V from 34 s to 4 s using varying current levels).

## 2. Design and Development

### 2.1. Three-Dimensional Modelling of the Structures

**Figure 1:**
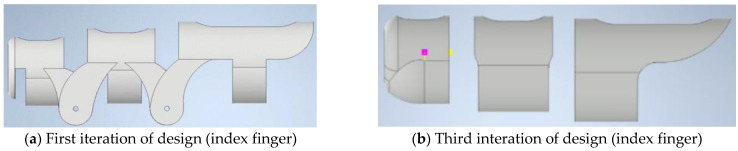

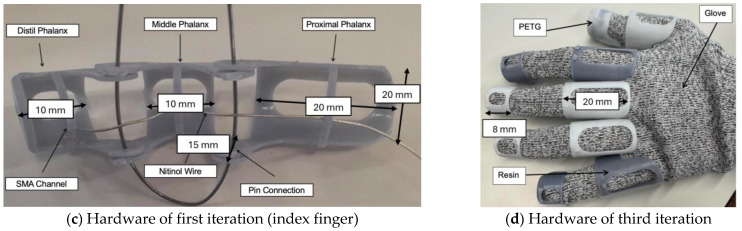
Three-dimensional modelling of finger (first iteration).

The design process began with modelling finger supports integrated into a glove to ensure both structural stability and proper alignment of SMA wire channels (Figure 1). Each finger section, proximal, middle, and distal phalanges, was sized for a snug, anatomically fit to the average hand sizes of users. The support structures for each finger are modelled sequentially with uniform pin connections for proper assembly. For example, for the index finger, dimensions are proximal (20 mm), middle (10 mm), and distal phalanges (10 mm), whereas the diameter is 20 mm. The overall weight of the system is around 100 gm, including the glove. A tapered shell was placed to replicate the natural contour of the human finger, and each segment included a 1.5 mm channel to house and insulate 1 mm Nitinol wire. Table 3 below summarises the key design parameters and calculations used in developing the hand exoskeleton. The values are based on standard anatomical measurements [38,39] to ensure a functional and ergonomic design. The shell thickness and gaps section defined the necessary clearance (1.5 mm) between the shell and the finger to optimise comfort, mobility, and durability. The shell is 2 mm thick to allow the user’s fingers to work as optimally as they can. The shell must be lightweight and strong enough not to break during use.

In the first iteration, the joint structure of each finger was designed based on the anthropometric data of average hand sizes of users. However, this feature was removed in the final iteration, as there is no need for additional joint-supporting structures. The design now relies on the user’s natural finger joints, with the SMA wire providing the necessary pulling force to assist in joint bending. The thumb was designed separately due to its unique function in gripping and pinching. It required extra space for its greater range of motion and was designed without pin joints, unlike the other fingers, to ensure better movement.

During the second design iteration, various adjustments were made to improve joint movements. The proximal phalanx was contoured to incorporate finger’s natural curvature, and the SMA channel was relocated to allow for free flexion movement. The thickness of the SMA channels was increased to prevent breakage during multiple uses and to improve functionality, resulting in smoother movement. To reduce slippage and improve finger movement, the design of the distal phalanx was adjusted. The third iteration focused on reducing the thickness of the SMA channels while maintaining strength. In the last iteration, mechanical pin connections were substituted with epoxy bonding to remove interference with the glove’s secondary layer and facilitate seamless integration of embedded electronics. The distal phalanges were also rounded to improve grip and comfort, resulting in a more functional and user-friendly device.

### 2.2. Nitinol Wire Setup

**Figure 2:**
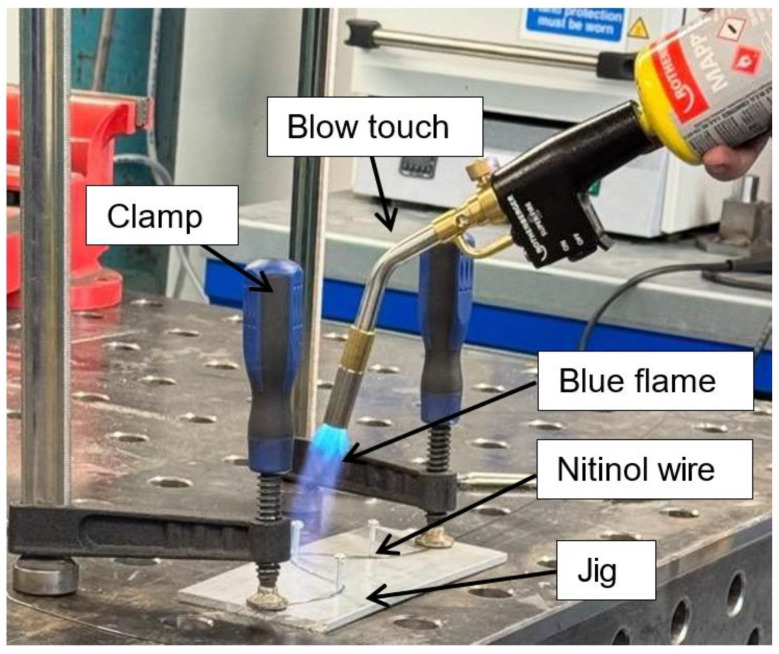
Blow torch based thermal excitation setup.

In the first setup, a clamp setup was created by taking a thick piece of scrap aluminium with two screws drilled at two points (Figure 2). After that, a Nitinol wire of 1 mm diameter and 150 mm length was fixed around those screws by using bolts. A blow torch is used to heat the wire above 600 °C from room temperature (25 °C) and quenched in cold water in a bucket. This process did not bend the metal; however, it only set a permanent shape. This confirmed that the wire was too thick and had thermosetting properties.

**Figure 3:**
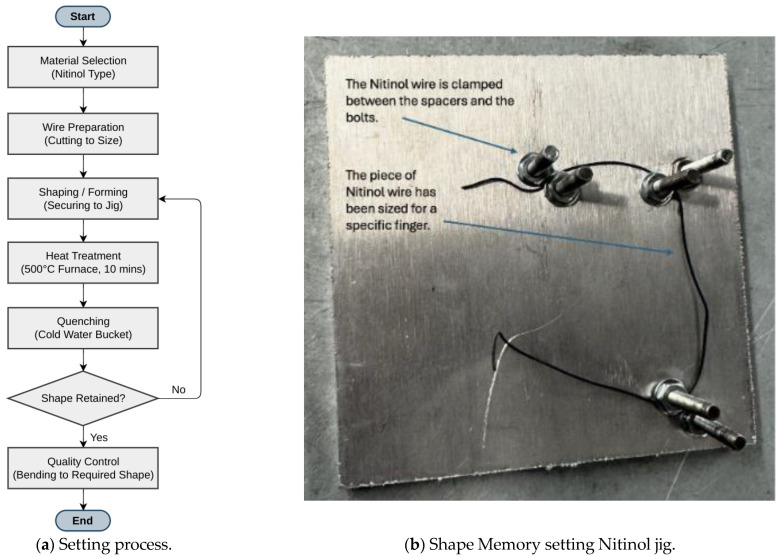
Final thermal stimulus jig.

Since the first setting was unsuccessful, the shape-changing phenomenon was evaluated through another controlled experimental setup (Figure 3). A thinner (0.3 mm diameter and 150 mm length) Nitinol wire was fixed into the custom jig. The jig had the shape of a closed fist. The wire was heated in an oven at 500 °C for 10 min from room temperature (25 °C). After heating, the wire was bent out of shape and then put in boiling water, where it immediately snapped back to its preset shape, confirming the shape-setting process was successful.

### 2.3. Prototype of the Device

**Figure 4:**
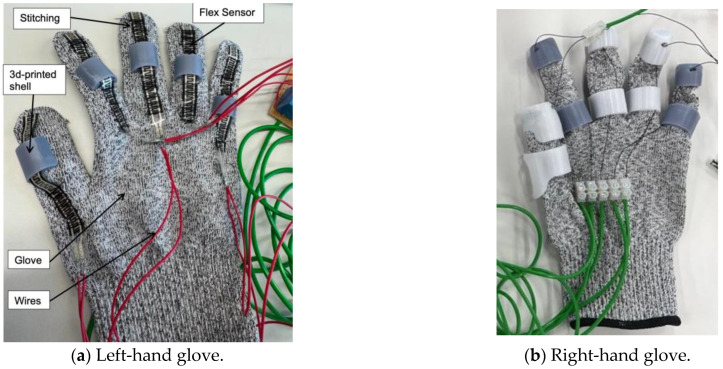
Prototype of the SMA embedded glove.

The setup features two gloves: one consists of flex sensors, and the other is integrated with Nitinol wire actuators (Figure 4). The flex sensors were stitched across the right-hand glove and further secured with adhesive at the fingertips to prevent slipping. The left-hand glove includes 3D-printed components attached to the glove using adhesive. Two distinct materials were used: resin for the grey parts and PETG for the white parts. This process was repeated to ensure all sensors were firmly in place. All components were designed and adjusted according to the specific anthropometric data of Table 3; therefore, the current design may not apply to all users. PETG and resin were selected after preliminary fabrication trials using multiple 3D-printing materials, including TPU. Although TPU offered good flexibility, the printed structures exhibited poor dimensional accuracy and insufficient rigidity. During fabrication trials, the thinner TPU segments frequently fractured after printing, making it unsuitable for this application. PETG provided an optimal balance between flexibility, strength, and thermal stability, with high impact resistance (7.5–8 kJ/m^2^), tensile strength of approximately 50 MPa, and a glass transition temperature around 80 °C. These properties ensured consistent dimensional integrity and mechanical reliability during repeated actuation cycles. Resin was selected for components requiring high surface accuracy and complex curvature, offering excellent dimensional precision, high stiffness (tensile strength ~60 MPa; modulus ~2.8 GPa), and a smooth surface finish. The combination of PETG and resin was particularly beneficial for this application: PETG provided robust mechanical support and thermal resistance around the SMA channels, while resin enabled lightweight, anatomically contoured sections with fine structural detail. Together, these materials delivered the required structural reliability, print precision, and thermal compatibility for the assistive finger segments. In the final iteration (Figure 4b), the thickness of the 3D-printed shell was optimised to maintain a minimal profile while ensuring sufficient structural integrity and proper guidance for the SMA wire. The final shell thickness of 2 mm provides finger articulation without interfering with its range of motion. However, this thickness also limits the heat transfer from the SMA wire during actuation. The material printing details are shown in Table 4.

### 2.4. Control Circuit

**Figure 5:**
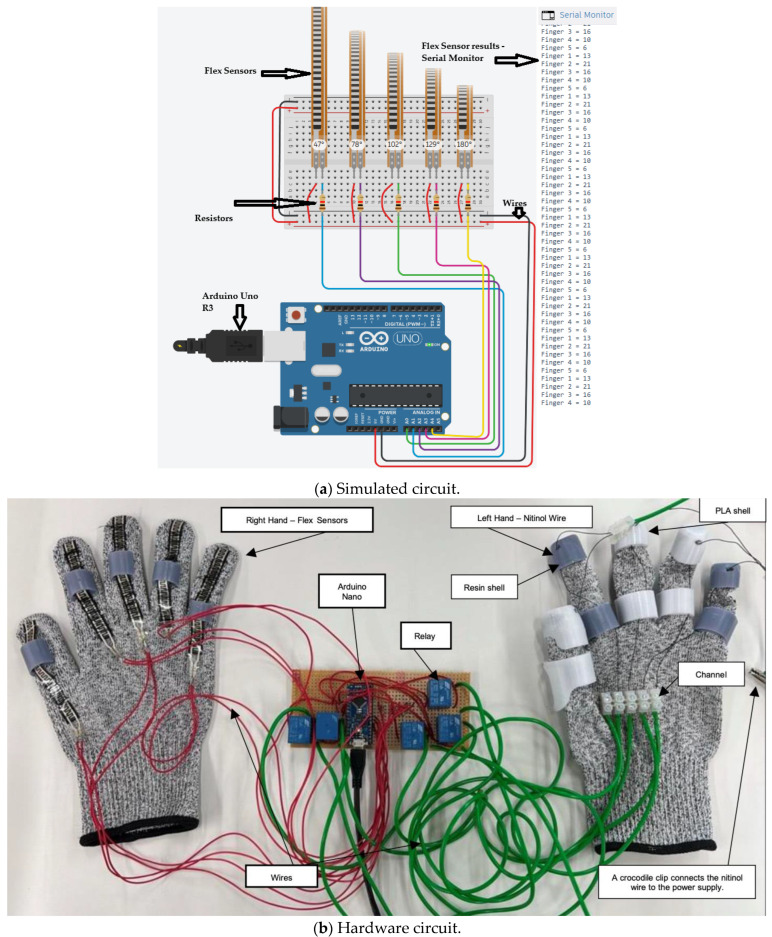
Embedded controller.

Two main input sensors, flex sensors and the MyoWare muscle sensor, are used for controlling and receiving feedback on user finger movement. The flex sensor was used to control each finger individually, allowing for precise and isolated finger movements. In contrast, the MyoWare sensor could only produce a full fist motion and could not control individual fingers. Based on these results, the flex sensor-based circuit was kept as a proof of concept for potential future development. One of the final circuit designs uses five flex sensors, each connected to a relay (Figure 5b). These relays switch the connection from the Arduino Nano to an external power supply, as the Arduino alone cannot provide enough current to activate the Nitinol wire.

Each piece of the Nitinol wire is assigned a threshold value in the code. When a flex sensor detects a bend that exceeds the assigned threshold (Arduino digital values 300), the corresponding relay switches on, allowing current to flow and move the Nitinol wire. The resistance of the flex sensor varies with the degree of bending; therefore, the voltage drop across the series resistor connected with the flex sensor changes accordingly (Figure 5a). The analogue input channel of the Arduino board provides a value between 0 and 1023, corresponding to an input voltage drop of 0 to 5 V DC. Hence, the analogue reading of 300 is equivalent to approximately 1.46 V DC. When the bend is no longer detected, the relay switches off, cutting the current and allowing the finger to return to its rest position. This control logic offered a mechanism suitable for use in rehabilitation and physical therapy, such as by executing pre-programmed movements that replicate the motion of an unaffected hand for the impaired one. The technical specifications of the components are shown in Table 5.

**Figure 6:**
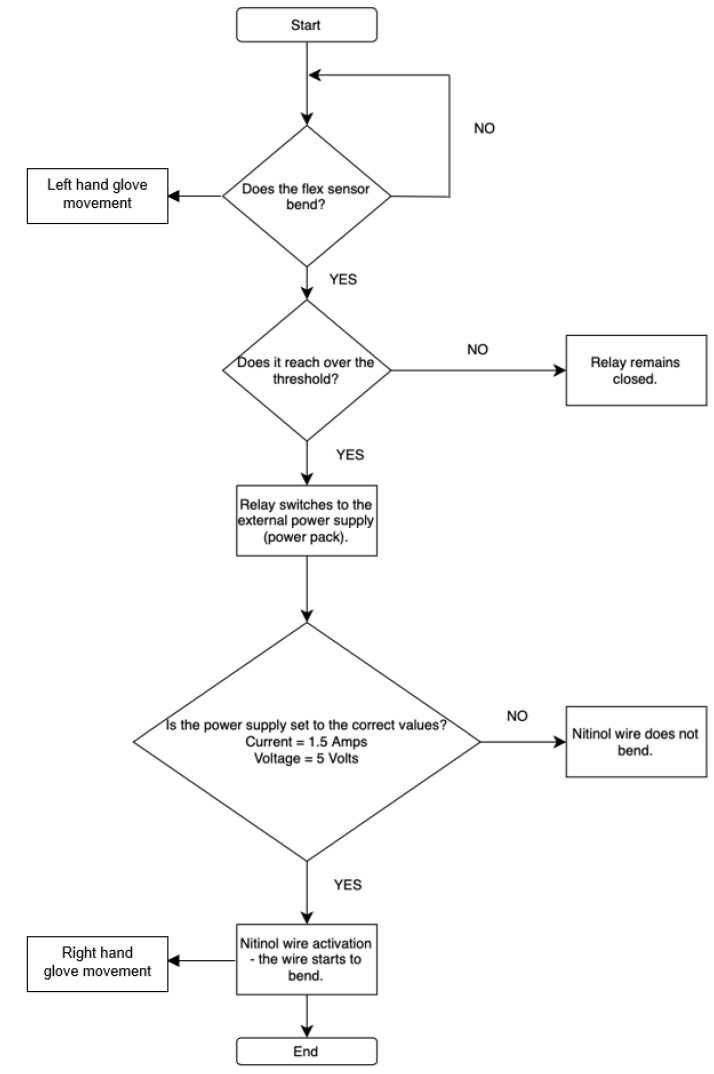
Flowchart for flex sensor setup.

An alternative control method was developed using the MyoWare EMG sensor (SparkFun Electronics, Niwot, CO, USA), using a counter-based system where a relay was activated once the muscle signal exceeded a predefined threshold. However, this method was less precise since the MyoWare sensor can misinterpret small, unintended muscle movements, such as tremors, making it less reliable than flex sensors. Although future improvements could involve mapping specific contraction ranges to individual fingers using EMG, this approach was not implemented in the proposed method. Instead, the focus remained on refining the flex sensor-based circuit, which provided more accurate and isolated finger control (Figure 6).

## 3. Results and Discussions

A series of tests was conducted to evaluate the activation time, thermal response, force capacity, range of motion, and finger trajectory in order to assess the effectiveness of the developed device.

### 3.1. Activation Test

**Figure 7:**
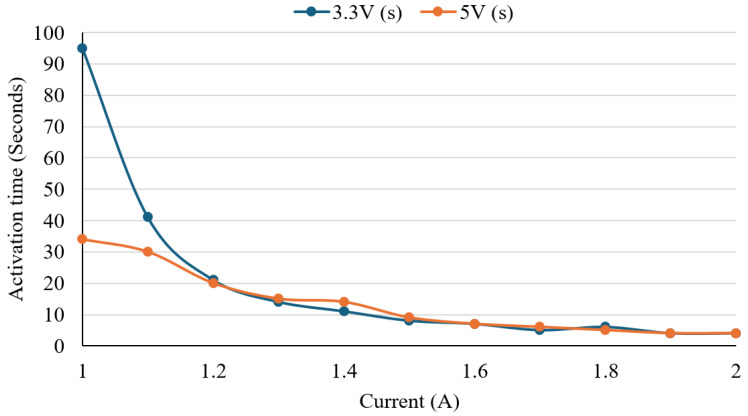
Effect of supply voltage on the activation time of Nitinol wire under varying current levels.

To evaluate the activation behaviour of the Nitinol wire and identify optimal electrical input conditions, a controlled heating experiment was conducted. The test served two main purposes, including determining the minimum current required for shape activation and evaluating how response time varied across different voltage and current combinations. The preset wire was heated using DC voltages of 3.3 V and 5.0 V, with current incremented from 1.0 A to 2.0 A in 0.1 A steps. At each step, the response time, defined as the duration from initial current application to full visual transformation, was recorded. Figure 7 presents a line graph of activation time versus current for both voltage levels. A clear inverse relationship was observed between current and activation time. At 3.3 V, activation time decreased from 95 s at 1.0 A to 4 s at 1.9–2.0 A. At 5.0 V, the activation time was faster at lower currents, starting at 34 s at 1.0 A and decreasing to 4 s by 1.9 A. Response times between voltage conditions became similar as the current increased beyond 1.5 A. An outlier was noted at 1.4 A, where 5.0 V produced a slower activation time than 3.3 V. Otherwise, both voltage conditions followed similar trends. The plateau after 1.5 A indicates that the advantages of response speed diminish as the current increases. The results suggest that both 3.3 V and 5.0 V can effectively activate the wire, with 5.0 V providing a slightly faster response at lower currents. The ultimate choice would rely on the device’s power availability and thermal safety requirements.

### 3.2. Thermal Response Analysis

**Figure 8:**
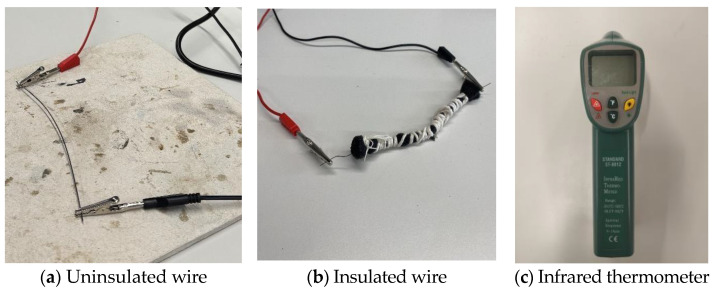
Thermal response analysis measuring setup.

A thermal response test was conducted to evaluate the effect of polyurethane foam insulation on the surface temperature of Nitinol wire during electrical activation (Figure 8). The wire was powered at 5.0 V, with current increasing from 1.0 A to 2.0 A in 0.1 A increments. The insulation layer is around 3 mm in diameter around the SMA wire for the experiment. For each current level, power was applied for 30 s, and surface temperature was recorded using an infrared thermometer (Standard Instrument, Dongguan, China). Tests were performed with and without foam insulation. A 30 s cooling interval was maintained between measurements to prevent thermal accumulation. The test was conducted with the following objectives in mind:

- To compare peak surface temperatures between insulated and non-insulated wire.
- To determine the thermal performance of the polyurethane foam.

**Figure 9:**
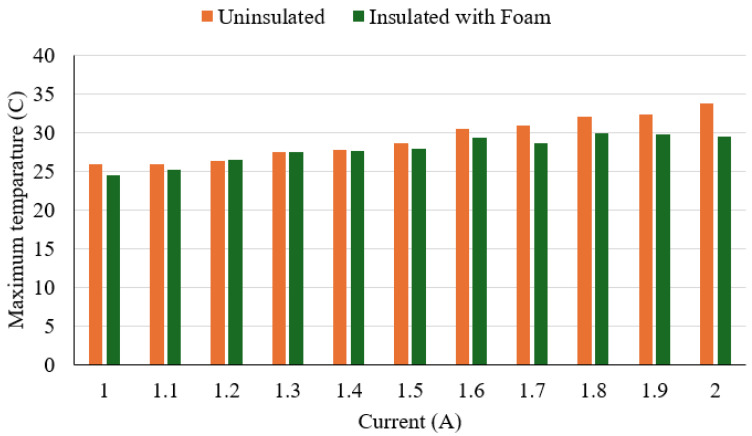
Effect of polyurethane foam on SMA temperature.

The experimental results show that the outer surface temperature increased steadily with current increases in both configurations (Figure 9). Without insulation, surface temperatures ranged from 26.0 °C at 1.0 A to 33.8 °C at 2.0 A. With insulation, temperatures ranged from 24.5 °C to 29.5 °C over the same current range. The maximum temperature reduction due to insulation was 4.3 °C at 2.0 A. The thermal difference between insulated and uninsulated conditions became more evident above 1.6 A, where insulation consistently maintained surface temperatures below 30.0 °C. These results indicate that the polyurethane foam provided moderate but consistent thermal dampening. Overall, the insulation effectively reduced surface temperature during wire activation. This can improve user safety by minimising thermal discomfort or risk of skin contact burns during device use.

### 3.3. Force Pulling Test

**Figure 10:**
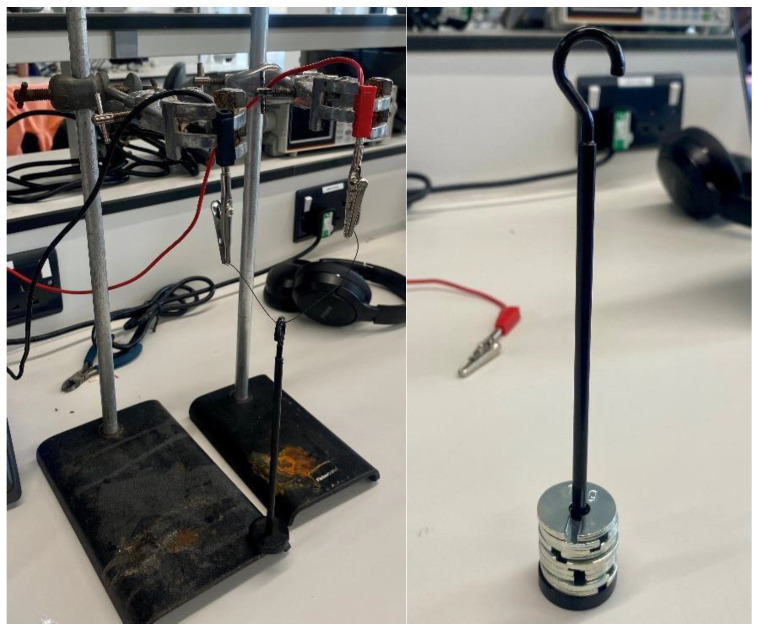
Force pulling test measuring setup.

A force-pulling test was conducted to determine the mechanical performance of the Nitinol wire under increasing loads (Figure 10). The experiment was conducted to identify the maximum load that the Nitinol wire (0.3 mm) could successfully lift under a stable electrical input, and to understand the relationship between the applied load and actuation performance. The wire was secured at both ends using a laboratory stand, with a central weight bar attached to apply incremental masses. The test started with the weight bar alone (0 g) to establish a baseline lift height, after which 10 g slotted weights were gradually added. The current was maintained at a constant 1.5 A throughout the test. For each applied load, the resulting vertical displacement from the base of the setup to the bottom of the weight bar was measured using a ruler.

**Figure 11:**
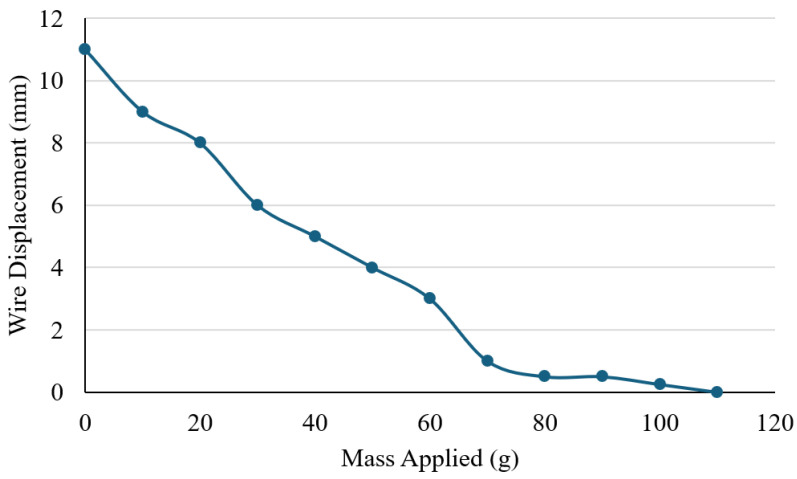
Effect of applied load on Nitinol wire actuation displacement.

Figure 11 illustrates the relationship between applied load and displacement. When unloaded (0 g), the wire achieved a maximum vertical movement of approximately 11 mm. As the load increased, displacement decreased steadily with each 10 g increment. At 100 g, only 0.25 mm of movement was recorded, and no measurable displacement occurred at 110 g, indicating that the wire was unable to lift the added weight. These results demonstrate a clear inverse relationship between load and displacement, with the wire’s maximum effective lifting capacity limited to around 100 g. Beyond this threshold, its mechanical output was insufficient to overcome the applied force. The findings provide important insight into the force generation limits of Nitinol actuators and help define their suitability for assistive hand mechanisms, where both motion and strength are essential for effective performance. However, it is acknowledged that 100 g may not be sufficient for performing higher loading daily activities. For higher load applications, additional Nitinol wires or wires with greater thickness will be used to increase the lifting strength.

### 3.4. Joint Range of Motion

**Figure 12:**
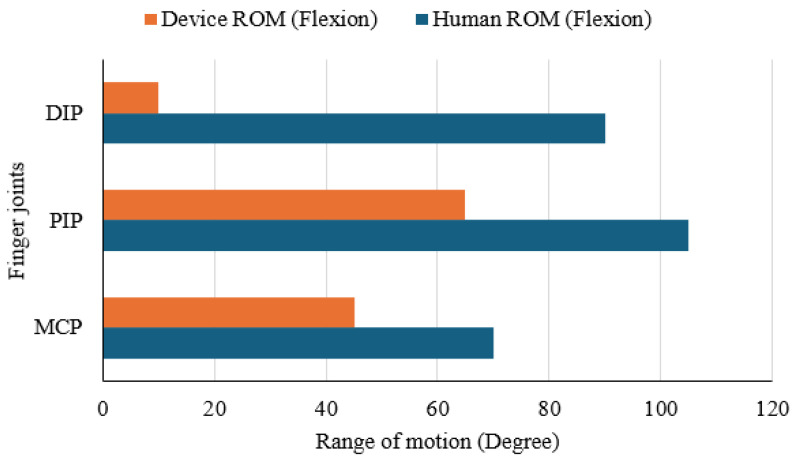
Comparison of finger joint range of motion: human hand vs. assistive device.

A joint range of motion (ROM) test was carried out to assess how effectively the Nitinol-actuated finger mimics natural human flexion. Flexion movement of a finger joint occurs primarily at three joints: the Metacarpophalangeal (MCP), Proximal Interphalangeal (PIP), and Distal Interphalangeal (DIP) joints. Angles of flexion movement at these points were measured for both a human index finger and the corresponding joints of the device using a standard goniometer. Figure 12 presents the comparison in a horizontal bar chart. The human finger reached flexion angles of approximately 70° at the MCP, 105° at the PIP, and 90° at the DIP. In contrast, the prototype achieved 45°, 65°, and 10° at the same joints, representing reductions of 35.7%, 38.1%, and 88.9%, respectively. These results indicate that the device can reasonably reproduce movement at the MCP and PIP joints but shows minimal articulation at the DIP joint. The restricted motion at the fingertip highlights a design limitation that should be addressed in future iterations to achieve a more natural bending profile.

### 3.5. Finger Trajectory Comparison

**Figure 13:**
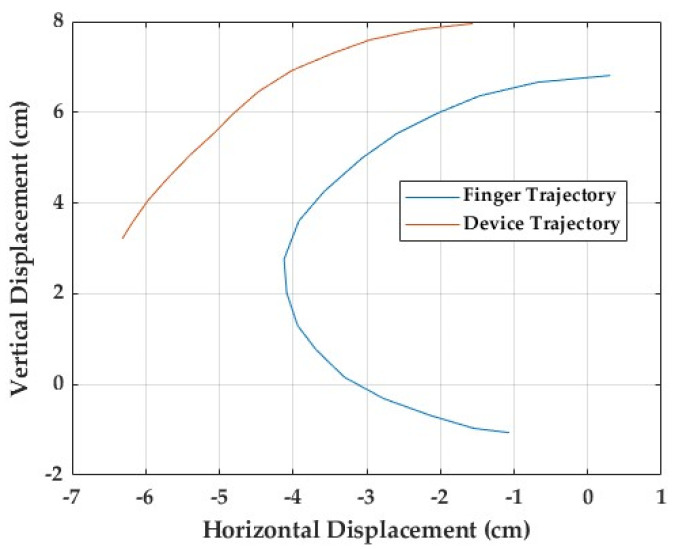
Finger flexion path analysis.

To evaluate the anatomical likeness of the device’s movement, a finger trajectory comparison test was conducted, focusing on the ring finger (Figure 13). A pen was fixed to the fingertip of a user’s hand. As the finger flexed naturally, the pen traced its movement path on a sheet of paper, providing a visual record of the finger’s motion for comparison with the device’s trajectory.

An identical procedure was used for the mechanical finger driven by the Nitinol wire, with a pen attached at the position corresponding to the fingertip. Both the human and device trajectories started from the same reference point, representing full extension at the metacarpophalangeal (MCP) joint. As flexion progressed, the human finger traced a smooth, continuous curve, reflecting the coordinated motion of all three joints. In contrast, the device produced a straighter, more linear and angular path, lacking the natural flow observed in the human finger. The recorded motion paths are presented in Figure 13. The human trajectory shows a wider, rounded arc with a brief hyperextension phase at the start of movement, while the device’s path is more constrained and rigid. The difference becomes increasingly evident toward the end of flexion, where natural finger curvature depends on bending in each individual joint to happen simultaneously. The difference between the two trajectories is around 37%. These findings suggest that, although the device generally follows the intended direction of motion, it does not fully replicate the natural curvature of the finger. The restricted range of flexion and limited joint coordination highlight key areas of improvement in the current mechanical configuration of the prototype.

### 3.6. EMG and Flex Sensor Test

**Figure 14:**
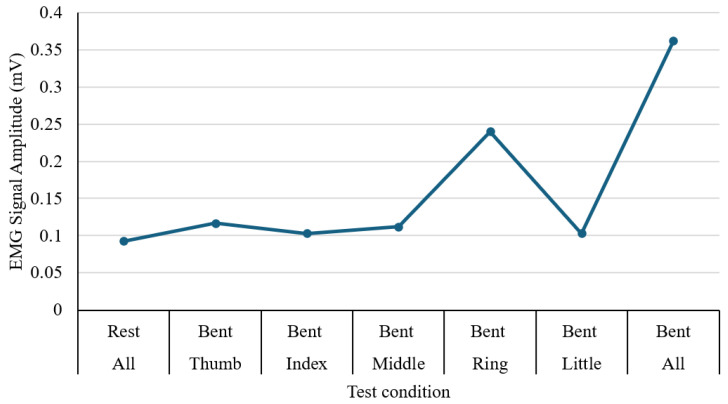
EMG signal amplitude during individual finger and hand movements.

The performance of muscle activity in the forearm during hand and finger movements is tested using an MyoWare Sensor 2.0, and the output was recorded through an Arduino interface connected to a laptop for real-time signal display. The experiment involved a sequence of actions, including resting, individual finger flexion (thumb, index, middle, ring, and little fingers), and a full fist clench. For each movement, the peak voltage amplitude was recorded in millivolts (mV), as illustrated in Figure 14. During rest, the signal remained low at approximately 0.093 mV, indicating minimal muscular activity. Among individual finger motions, the ring finger generated the strongest signal (0.240 mV), while the index and little fingers produced the lowest values (0.103 mV). The full fist clench showed the highest overall amplitude at 0.362 mV, reflecting simultaneous activation of multiple muscle groups. These results confirm that the EMG sensor effectively differentiates between varying degrees of muscle engagement. The apparent differences in signal magnitude across finger gestures highlight the potential of EMG input as a reliable control method for assistive hand or rehabilitation devices.

**Figure 15:**
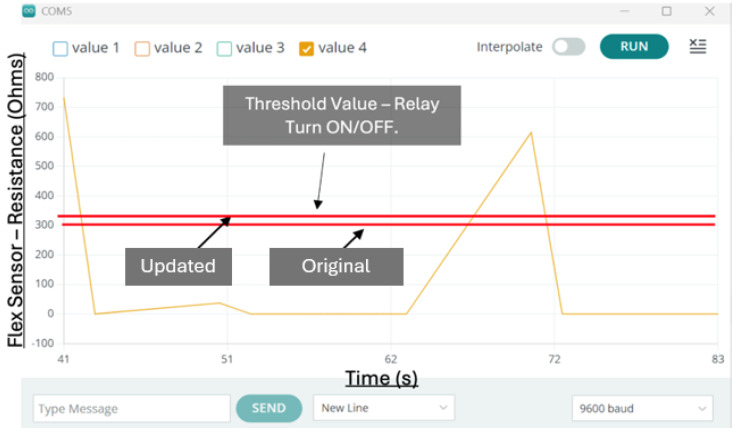
Data from flex sensor.

Figure 15 illustrates the input signal received by the Arduino from the flex sensor. The signal maintains a baseline above zero, as values below this point correspond to the hand’s natural rest state and its anatomical limits. When the sensor reading rises beyond 300, the relay is triggered, activating contraction of the Nitinol wire. A programmed delay of 500 milliseconds was applied to regulate the actuation and prevent abrupt motion. Once the signal falls below the threshold, the relay disengages, allowing the wire to return to its relaxed state. After integrating the glove shell, the activation threshold was refined to 350 to account for the sensor’s altered response characteristics.

## 4. Discussions

The developed prototype consists of a dual-glove system designed for hand rehabilitation. The right-hand glove contains flex sensors that detect finger bending, while the left-hand glove is fitted with Nitinol wires that contract when activated. When the flex sensor on the right glove bends beyond a set threshold, the corresponding wire on the left glove contracts. Although visible finger motion was minimal, contraction of the wire was noticeable near the palm section of the glove. As Nitinol cannot be easily soldered due to its oxide layer, electrical connections were made using crocodile clips. Small openings were created at the glove’s fingertips to route the wires securely. The thumb, index, and middle fingers were linked to one actuation channel, while the ring and little fingers shared another. Each channel was powered separately to allow independent operation. During testing, the trigger threshold was fine-tuned to a value of 350 to compensate for irregularities caused by the flex sensors against the glove fabric.

Experimental results for activation speed, force, and temperature behaviour were encouraging and indicated potential for rehabilitation use. Nevertheless, there is scope for improvement, particularly in increasing joint movement range and producing a smoother, more natural finger path. The Nitinol wire was tested at supply voltages of 3.3 V and 5.0 V with currents between 1.0 A and 2.0 A. Response time decreased as the current rose, with 5.0 V providing faster activation at lower currents, though performance gains saturated above 1.5 A. Thermal tests showed that polyurethane foam insulation lowered the wire’s surface temperature by about 4.3 °C and kept it below 30 °C at currents above 1.6 A, improving user safety during longer use.

Force–displacement analysis demonstrated that at 1.5 A, the actuator could lift a 100 g load, achieving 11 mm movement under no load and 0.25 mm at 100 g. No motion occurred at 110 g, confirming that the design cannot yet generate higher lifting forces. EMG measurements taken during hand motion showed clear variations in electrical activity: resting potential was around 0.093 mV, the ring finger produced a peak of 0.240 mV, and a full fist clench reached 0.362 mV. These patterns indicate that muscle signals can effectively be used to detect and classify hand gestures for potential device control. Hence, integration of EMG sensing could be a valuable direction for future work in terms of detecting finger movement and controlling it.

Although results were promising, the glove’s range of motion remained limited, particularly at the distal joints. This suggests that the current mechanical structure and the way the Nitinol wire is shaped restrict full finger articulation. The fingers also tended to move linearly rather than following the curved, natural motion of the human hand. This lack of coordinated joint movement reduced anatomical accuracy and overall realism. Temperature measurements could not be continuously recorded because of placement constraints, though intermittent checks are performed. The long-term thermal safety remains an important consideration, and future research should include reliable temperature sensors and an easy method for replacing worn wires.

## 5. Conclusions

This article explores the development of a novel assistive hand device using SMA (Nitinol wire). The system shows promise for early-stage stroke therapy and other rehabilitation applications of the affected hand, allowing mirrored movements using healthy hands that guide recovery through exercises. However, there are a few structural limitations that impact the device’s overall performance. For example, thick wire will support a higher loading capacity while also bringing rigidity in terms of movement. It has been found that wire thickness over 1 mm tends to become rigid when heated, whereas thinner wires remain flexible during the assistive movement and can easily return to their original shape. Hence, it is crucial to find a balanced approach to support both loading capacity and flexibility in joint movement. The developed prototype has a relatively low loading capacity of up to 1 N, whereas a similar device can produce 5 N. However, it offers other advantages, such as thermal management, compliance, and linearised control over joint articulation, which can support exercises for hand and finger movements. The experimental results suggest that the device could be beneficial in supporting lower actuation forces while maintaining flexibility. Areas identified for improvement include increasing the output force by utilising multiple Nitinol wires and enhancing the overall structural integrity.

The current dimensions are determined for an average hand-sized user; however, it could be customised and modified based on other hand sizes. To avoid connection issues between the Nitinol wire and other interfacing circuits, a fuse terminal block can be used, which not only allows high current flow through the circuit but also enables the Nitinol wire to be tightly attached to the other part, even under heated conditions. Sensor fusion of EMG and flex sensors can be utilised to control the joint movements in a more precise and linear way. Smart SMA wire is also a potential option that could be helpful in combination with other materials or passive actuators, such as springs or rubber bands, to limit the energy consumption due to high current draw during operations. Other advanced techniques can be used to support thermal management. More experimental analysis should be performed to understand usability and wearability for long-term use.

## Figures and Tables

**Table 1:** Academic and Research Devices Using SMA.

Study/Device	Design and Actuation	Key Features and Performance
Hybrid Anthropomorphic Prosthetic Hand [19]	Tendon-driven fingers actuated by antagonistic SMA wires; EMG-based PWM control	3 active DOF; natural finger motion
Soft 3D-Printed Hand (Coiled SMA) [16]	TPU 3D-printed hand, actuated by coiled SMA	Lightweight (235 g); 425 mm; grasp 55–81 mm; max 133 g load; 7.82 W power
Soft Five-Fingered SMA Hand [21]	SMA wires (100 μm); welded in bundles for higher force	Flexible, silent, customizable; grasps various objects; measured phalanx displacement
Linear SMA (Nitinol) Prosthesis [17]	Nitinol-based actuators with “two-link transmission”	Power grip: 16.6 N; pinch: 4.4 N; fast actuation (0.06 s heating); accurate (error < 1°)
Hand Movement Device (HMD) [24]	SMA-driven compact device for ADLs	MCP: 45°, PIP: 62°, DIP: 71°; grip force: 40 N
Flexible SMA Actuators (Exoskeleton) [23]	SMA wires with nonlinear and model-free control	Wearable exoskeleton; improved adaptability and comfort
Soft Wrist Assist (SWA) [22]	SMA wires; wearable wrist device	Force: 10 N; contraction: 40%; torque: 1.32 Nm; lightweight (151 g)
Multifunctional Prosthetic Hand [20]	3D printed, tendon-driven, SMA actuated; tactile sensors	Small, lightweight, quiet; resistance-based feedback control

**Table 2:** Commercial Assistive/Rehabilitation Devices.

Device	Function	Features	Actuation	Price
XFT Hand Rehab System [25]	Post-injury rehab	Muscle stimulation; improves blood flow, coordination; portable	Pneumatic	£899.00
Perfect TENS + iGlove [26]	Pain relief (chronic, musculoskeletal, acute)	Conductive fabric glove; dual-channel TENS	Electrical stimulation	£66.60
SaeboGlide Plus [27]	Arm/hand rehab	Sleeve-assisted guided exercises, for long-term use, operates by Passive elastic tensioners	Spring assisted	£78.00
Radial Nerve Palsy Brace Splint [28]	Stroke/nerve recovery	Elastic finger strips; wrist/finger support	Elastic band	£49.95
EMFOCU Robotic Rehab Gloves [29]	Stroke rehab	Finger rotation, traction, mirror training; LED display	Pneumatic	£102.00
Advanced Rehab Robot Gloves (ixorelia) [30]	Stroke and neuro rehab	Smart therapy glove, mirror glove, training ball	Pneumatic	(Not listed)
Rehabilitation Robot Glove (Gear Tech) [31]	Stroke recovery	9 speeds, 3 modes; breathable fabric; 8 h battery	Pneumatic	£74.99
XTENSOR [32]	Grip/strength training	Reverse grip exerciser; finger bands; wrist strap	Elastic band	£38.00

**Table 3:** Anthropometric data for developing the device.

Component	Parameter	Value
Finger Lengths (Unisex, Midpoint Values)		
Thumb	Total Length	63.5 mm
	Proximal Phalanx	35 mm
	Distal Phalanx	28.5 mm
Index Finger	Total Length	74.5 mm
	Proximal Phalanx	40 mm
	Middle Phalanx	20 mm
	Distal Phalanx	14.5 mm
Middle Finger	Total Length	82.5 mm
	Proximal Phalanx	42.5 mm
	Middle Phalanx	23 mm
	Distal Phalanx	17 mm
Ring Finger	Total Length	77.5 mm
	Proximal Phalanx	40 mm
	Middle Phalanx	20 mm
	Distal Phalanx	17.5 mm
Little Finger	Total Length	62.5 mm
	Proximal Phalanx	35 mm
	Middle Phalanx	15 mm
	Distal Phalanx	12.5 mm
Finger Diameter (Tapered Design)		
Thumb	Proximal	24 mm
	Distal	20 mm
Index	Proximal	19 mm
	Middle	17 mm
	Distal	14 mm
Middle	Proximal	21 mm
	Middle	19 mm
	Distal	16 mm
Ring	Proximal	19 mm
	Middle	17 mm
	Distal	14 mm
Little	Proximal	16 mm
	Middle	14 mm
	Distal	12 mm

**Table 4:** Material printing parameters.

Parameter	Details
Printer Used	Ultimaker S5 (FDM—PETG material) Formlabs Form 3 (SLA—Resin material)
Materials	PETG: Durable, impact-resistant, slight flexibility Resin: High-detail, smooth surface, rigid
MYOWARE 2.0	Collect EMG data for finger movement
Layer Height	PETG (FDM): 0.1 mm Resin (SLA): 0.05 mm
Nozzle Size (FDM)	0.4 mm
Infill (FDM)	20–50%
Printing Technologies	FDM (Fused Deposition Modelling)—for strong prototypes SLA (Stereolithography)—for fine, high-detail parts

**Table 5:** Technical specifications of components.

Component	Function	Technical Features	Dimension and Mass	Price
Arduino Nano board	Collect data from the flex and EMG sensor and control the relay for SMA actuation	Microcontroller: ATmega328 Operating Voltage: 5 V SRAM 2 KB Clock Speed: 16 MHz EEPROM 1 KB DC Current: 20 mA Input Voltage 7–12 V Power Consumption: 19 mA	Dim: 18 × 45 mm Weight: 7 g	£25.00
ZD10-100 Flex Bend Sensor	Collect the bending angles of finger movement in terms of resistance variation	Range: 0~500 g Thickness: less than 0.25 mm Response Point: less than 20 g Test Voltage: DC 3.3 V Response Time: less than 10 ms Recovery Time: less than 15 ms Working Temperature: −20 °C~60 °C	Dim: 110 × 10 × 25 mm Weight: 3 g	£5.00
MYOWARE 2.0	Collect EMG data for finger movement	Supply Voltage: 3.3 V or 5 V Input Impedance: 800 Common Mode Rejection Ratio (CMMR): 140 dB Ideal Gain Equation: Raw (RAW): G = 200 Rectified (RECT): G= 200 Envelope (ENV): G = 200 × R/1 kOhm	Dim: 37.57 mm × 35.90 mm	£32.00
Relay	Control the SMA wire activation from the Arduino board	Coil Voltage: 5 V DC Max. Operating Voltage: 277 V AC/28 V DC Rated Voltage: 3 to 48 V DC Nominal Operating Power: 0.36 W, 0.45 W Contact: 7 A 240 V AC, 10 A 125 V AC, 10 A 28 V DC SPDT type 5-pin terminals	Dim: 19 × 15 × 15 mm	£2.00

## Data Availability

The original contributions presented in this study are included in the article. Further inquiries can be directed to the corresponding author.
